# Association of Preoperative Imaging and Surgical Delay with Hemorrhagic Mortality in Abdominal Trauma: A Retrospective Multicenter Study

**DOI:** 10.3390/jcm14197020

**Published:** 2025-10-03

**Authors:** Juhong Park, Youngmin Kim, Hangjoo Cho, Giljae Lee, Junsik Kwon

**Affiliations:** 1 Department of Surgery, Division of Trauma Surgery, Ajou University School of Medicine, Suwon 16499, Republic of Korea; doginzoo@aumc.ac.kr; 2 Department of Trauma Surgery, Gachon University Gil Medical Center, Incheon 21565, Republic of Korea; skyofmay@gilhospital.com; 3 Department of Trauma Surgery, Uijeongbu St. Mary’s Hospital, College of Medicine, The Catholic University of Korea, Seoul 06591, Republic of Korea; surgeryman@catholic.ac.kr

**Keywords:** abdominal trauma, hemorrhagic shock, emergency laparotomy, surgical delay, trauma resuscitation, time-to-intervention, preoperative computed tomography

## Abstract

**Background:** Surgical delay in abdominal trauma with hemorrhage is a leading cause of preventable death, yet the precise time threshold for adverse outcomes remains uncertain. This study examined the association between emergency department (ED)-to-operating room (OR) time and hemorrhagic mortality and evaluated the impact of preoperative computed tomography (CT). **Methods:** We retrospectively analyzed patients ≥15 years old who underwent emergency laparotomy for abdominal trauma at two Level I trauma centers in South Korea (2016–2023). The primary outcome was hemorrhagic death, adjudicated by a multidisciplinary review panel. Multivariable and segmented logistic regression was used to assess the association between ED-to-OR time and mortality. The effect of preoperative CT was evaluated using inverse probability of treatment weighting (IPTW). **Results:** Among 414 patients, 71 (17.1%) died from hemorrhage. Each 1-min increase in ED-to-OR time was associated with 1.8% higher odds of hemorrhagic death (adjusted OR = 1.018; 95% CI, 1.007–1.030). Segmented regression identified a changepoint at 91 min (bootstrap 95% CI, 62.0–97.6), beyond which mortality risk rose sharply. Preoperative CT was performed in 27.5% of patients and was associated with a mean surgical delay of over 30 min. After IPTW adjustment, CT use was not significantly associated with hemorrhagic death (14.3% vs. 10.3%, *p* = 0.542). **Conclusions:** Longer ED-to-OR intervals were associated with increased hemorrhagic mortality, particularly beyond approximately 90 min. Although preoperative CT contributed to procedural delay, it was not independently associated with worse outcomes when selectively used in stable patients. These findings represent observational associations in current practice rather than causal effects, underscoring the importance of minimizing surgical delay while cautiously considering CT in appropriate patients.

## 1. Introduction

Trauma is a leading cause of death worldwide, and uncontrolled hemorrhage accounts for nearly one-third of all trauma fatalities. Hemorrhage is a major cause of potentially preventable death and can manifest rapidly; the median time from the onset of hemorrhagic shock to death is approximately 2 h [1]. In cases of abdominal trauma, internal bleeding can quickly lead to hemodynamic collapse, making prompt surgical control essential to prevent exsanguination.

Major trauma guidelines prioritize rapid hemorrhage control; for example, the American College of Surgeons Committee on Trauma and the Eastern Association for the Surgery of Trauma recommend minimizing the time from injury to surgical intervention in cases of severe hemorrhage, reflecting the “golden hour” concept that early control improves survival [2]. Several studies have quantitatively examined the impact of surgical delay on mortality. Clarke et al. reported that delays beyond 90 min in abdominal hemorrhage control were associated with significantly increased mortality [3]. Malinoski et al. demonstrated that a diagnostic delay of approximately 5 h increased the risk of death in patients with blunt hollow viscus injury [4], while Fakhry et al. found that delays exceeding 8 h were associated with higher morbidity and mortality in small bowel injury [5]. More recently, Barbosa et al. showed that among trauma patients requiring emergency laparotomy, each 10-min delay from emergency department (ED) arrival to the operating room (OR) was associated with a 50% increase in the odds of death [6]. These findings underscore the principle that “time is blood” in hemorrhagic shock, yet quantitative data on ED-to-OR timing remain limited, highlighting the need for further investigation.

In unstable patients, preoperative CT may substantially delay surgical intervention, yet it is sometimes performed even when immediate laparotomy is indicated. A large trauma registry study found that hypotensive patients who underwent abdominal CT before emergency laparotomy experienced significantly longer delays and much higher mortality rates (45% died vs. 30% with immediate surgery), with preoperative CT independently associated with 70% higher odds of death [7].

This study was designed to address this knowledge gap. We hypothesize that among trauma patients requiring emergency abdominal surgery, a shorter ED-to-OR time is associated with lower hemorrhage-related mortality and that obtaining a preoperative CT scan contributes to delays and increases the risk of hemorrhagic death. By quantitatively evaluating ED-to-OR intervals and preoperative CT use, this study aimed to evaluate observational associations between surgical timing, imaging decisions, and outcomes. Given the retrospective design, the findings should be interpreted as associations rather than causation.

## 2. Materials and Methods

### 2.1. Study Design and Data Sources

This study was conducted as part of the Multicenter Initiative to Reduce Hemorrhagic Mortality in Abdominal Trauma through Timely Emergency Surgery, sponsored by the Korean Association for Trauma Surgery. We retrospectively analyzed data from patients with severe abdominal trauma collected at two Level I trauma centers in South Korea between 2016 and 2023. The research data were obtained from the Korea Trauma Data Bank (KTDB), a national trauma registry that contains standardized clinical information from patients treated at regional trauma centers across Korea and is used for research and quality improvement purposes.

### 2.2. Patient Selection and Data Collection

**Figure 1:**
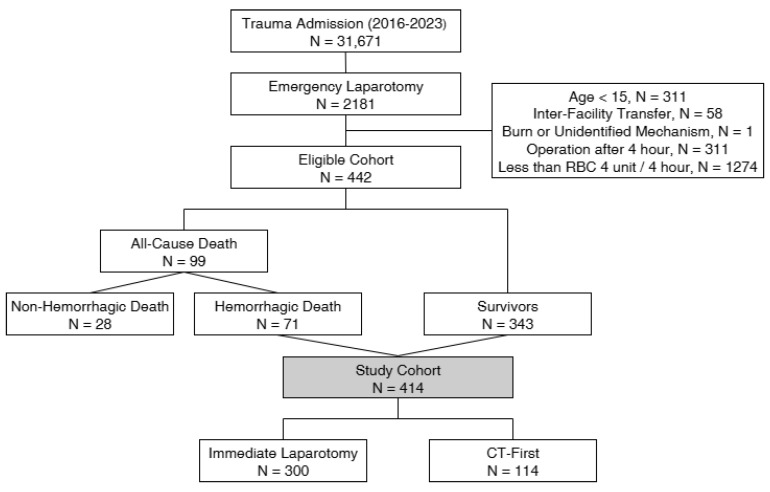
Patient selection flowchart.

Inclusion criteria were patients aged 15 years or older who were directly transferred from the scene and underwent emergency abdominal surgery after ED arrival. Exclusion criteria included patients with burns or an indeterminate injury mechanism, those with more than 4 h from ED arrival to surgery initiation, or those receiving fewer than 4 units of packed red blood cells (PRBCs) within 4 h of ED arrival. The traditional definition of massive transfusion is the administration of ≥10 units of PRBCs within 24 h. However, because this study specifically targeted patients who underwent early laparotomy for ongoing hemorrhage, we adopted an earlier time frame to better capture patients with clinically significant early bleeding. The Korea Trauma Data Bank (KTDB) provides transfusion records in 4-h increments, and we therefore defined our cohort as patients who received ≥4 units of PRBCs within the first 4 h after ED arrival. While previous studies have proposed alternative thresholds to mitigate survival bias, there is currently no universally accepted standard or consensus definition for early transfusion-based inclusion [8,9,10]. Accordingly, we acknowledge that our 4-h/4-unit cutoff is not universally standardized and may represent a limitation. The selection process for the study cohort is illustrated in Figure 1.

### 2.3. Criteria for Preoperative CT and Immediate Surgery

All patients underwent initial resuscitation and primary survey according to Advanced Trauma Life Support (ATLS) protocols. Preoperative CT was performed only in patients who were hemodynamically stable or stabilized (systolic blood pressure ≥90 mmHg without escalating vasopressor or transfusion requirements). Immediate laparotomy was undertaken in patients who remained hemodynamically unstable despite initial resuscitation (e.g., persistent hypotension with escalating transfusion or vasopressor requirements), either when intra-abdominal hemorrhage was clinically suspected or when a focused assessment with sonography for trauma (FAST) examination was positive. Because of the retrospective design, individual clinician judgment may also have influenced these decisions, which was not fully captured in the records.

### 2.4. Definition of Hemorrhagic Death

At both participating trauma centers, trauma-related deaths were routinely evaluated through a multidisciplinary panel review process, a methodology widely applied in trauma quality improvement research [11]. The panel included general surgeons, emergency physicians, thoracic surgeons, and neurosurgeons. For each case, standardized summaries were prepared containing prehospital and in-hospital information, including initial vital signs, imaging (FAST and CT), operative and interventional reports, transfusion records, and subsequent clinical course. The panel determined the primary cause of death by consensus. Patients were classified as hemorrhagic deaths if uncontrolled bleeding was judged to be the primary cause.

### 2.5. Study Variables and Primary Outcome Definition

The following variables were collected and analyzed: patient demographics (age, sex); injury characteristics, including mechanism of injury, the Abbreviated Injury Scale by body region (AIS) and the Injury Severity Score (ISS); physiologic parameters at ED arrival, including systolic blood pressure, pulse rate, the Glasgow Coma Scale (GCS), and the Revised Trauma Score (RTS); and transfusion requirements, defined as the number of PRBC units transfused within the first 4 h and within 24 h of ED arrival. The primary exposure was the use of preoperative CT versus immediate laparotomy. The primary outcome was hemorrhagic death, adjudicated by the multidisciplinary mortality panel. Secondary outcomes included all-cause in-hospital mortality and differences in ED-to-OR time between patients undergoing preoperative CT and those undergoing immediate laparotomy. ED-to-OR time was defined as the interval in minutes from ED arrival to surgical incision.

### 2.6. Statistical Analysis

Continuous variables were summarized as mean ± standard deviation or median (interquartile range). Group differences were compared using Student’s *t*-test or the Mann–Whitney U test for normally and non-normally distributed variables, respectively. Categorical variables were presented as frequencies and percentages and were analyzed using either the chi-square test or Fisher’s exact test, as appropriate. To identify risk factors for hemorrhagic death, multivariable logistic regression analysis was performed to calculate adjusted odds ratios (ORs), controlling for potential confounders.

To compare ED-to-OR times based on preoperative CT status, inverse probability of treatment weighting (IPTW) was applied with an average treatment effect (ATE) target. Propensity scores were estimated from a logistic regression model including the following baseline covariates: age, systolic blood pressure, pulse rate, GCS, RTS, AIS by body region, ISS, and 4-h transfusion volume. No automated variable selection procedures were used. Covariate balance was assessed using standardized mean differences (SMDs) and by visually inspecting the overlap of propensity score distributions. An adequate balance was prespecified as an SMD <0.10. Standardized mean differences before and after weighting were illustrated in a Love plot (Supplementary Figure S1). To address residual imbalances, we additionally performed a doubly robust augmented inverse probability weighting (AIPW) analysis including the same covariates. The ‘weighted n’ values represent the sum of weights rather than actual patient counts. Depending on propensity score distribution and scaling, these may appear larger or smaller than the original sample size.

For the changepoint model, we applied a segmented logistic regression to examine whether delays in ED-to-OR time were linked to hemorrhagic mortality. This approach allowed the slope of risk to change after a data-driven threshold. Segmented logistic models were iteratively fitted across candidate ED-to-OR cutoffs, and the optimal changepoint was estimated by maximum likelihood. Model fit was compared with a single-slope model using the likelihood ratio test. The analysis was adjusted for age, systolic blood pressure, GCS, ISS, and 4-h RBC transfusion. Confidence intervals for the changepoint were derived using bootstrap resampling and further supported by profile-likelihood evaluation to provide robust uncertainty estimates. For the outcome models (multivariable and segmented logistic regression), assumptions were evaluated by testing linearity of continuous predictors in the logit, examining multicollinearity with variance inflation factors (VIF), identifying influential observations, and assessing overall model calibration.

All statistical analyses were performed using Python version 3.13.7 (Python Software Foundation, Wilmington, DE, USA), employing functions from the statsmodels, scikit-learn, and Matplotlib packages (version 3.9.2; https://matplotlib.org/). All tests were two-sided, and statistical significance was defined as *p* < 0.05.

### 2.7. Ethical Considerations

This study utilized anonymized data obtained from a publicly available database. Ethical review and approval were provided by the Institutional Review Board of Ajou University Hospital (approval No. AJOUIRB-DB-2025-167; approval date: 31 March 2025), which waived the requirement for patient informed consent. All methods and reporting adhered to the STROBE (Strengthening the Reporting of Observational Studies in Epidemiology) guidelines. The complete STROBE checklist is provided in Supplementary Materials.

## 3. Results

### 3.1. Comparison Between Hemorrhagic Death and Survivors

Among the 414 patients included, all-cause death occurred in 99 (23.9%) including 71 (17.1%) hemorrhagic deaths. Clinical characteristics are summarized in Table 1. Gender distribution (male: 78.9% vs. 77.8%) and mean age (49.0 vs. 48.5 years) were similar between the two groups (*p* = 0.966 for sex; and *p* = 0.813 for age).

Blunt trauma was more frequent in the hemorrhagic death group (90.1% vs. 78.7%, *p* = 0.041). The pulse rate at arrival was significantly higher in the hemorrhagic death group (90.4 vs. 82.1, *p* = 0.022). The GCS and RTS values were significantly worse in the hemorrhagic death group (GCS: 7 vs. 14, *p* < 0.001; RTS: 9.3 vs. 10.8, *p* < 0.001).

The hemorrhagic death group had a higher abdominal AIS (4.1 ± 1.1 vs. 3.5 ± 0.9, *p* < 0.001), indicating more severe abdominal injury. In contrast, head AIS was lower in the hemorrhagic death group (0.3 vs. 1.0, *p* < 0.001), suggesting less severe head injuries. The ISS was numerically higher in the hemorrhagic death group compared to survivors, but this not statistically significant (32.6 vs. 30.2, *p* = 0.170).

The mean volume of RBC transfusion within 4 h of ED arrival, an indicator of initial hemorrhage severity, was significantly higher in the hemorrhagic death group than in survivors (18.0 ± 7.0 vs. 10.3 ± 5.8 units; *p* < 0.001). Similarly, the 24-h transfusion volume was greater in the hemorrhagic death group (23.1 vs. 12.4 units, *p* < 0.001).

The mean ED-to-OR time was also longer in the hemorrhagic death group (62.3 ± 31.9 min) compared to survivors (56.5 ± 26.2 min), but the difference was not statistically significant. (*p* = 0.107).

### 3.2. Multivariable Analysis of Factors Associated with Hemorrhagic Death

Multivariable logistic regression analysis, adjusting for major clinical variables, was performed to identify predictive factors for hemorrhagic death (Table 2). The results showed that ED-to-OR time delay was independently associated with higher odds of hemorrhagic death (OR, 1.018, 95% CI, 1.007–1.030; *p* = 0.002), indicating that each 1-min delay in the ED-to-OR time increased the odds of hemorrhagic death by approximately 1.8%. Although the crude comparison in Table 1 did not reach statistical significance, this discrepancy likely reflects confounding, as more unstable patients underwent expedited surgery. After adjustment, the independent association between surgical delay and hemorrhagic mortality became apparent. Initial 4-h RBC transfusion volume was strongly associated with hemorrhagic death and was considered as a surrogate of initial hemorrhage severity, not as a causal exposure (OR, 1.176 per 1-unit increase; 95% CI, 1.118–1.237; *p* < 0.001).

Conversely, the GCS score showed a negative correlation, with an OR of 0.799 (95% CI, 0.739–0.864; *p* < 0.001), indicating that a higher initial GCS score was associated with a decreased risk of hemorrhagic death. Other variables—such as age, ISS, and initial systolic blood pressure—were not statistically significant predictors (all *p* > 0.05).

### 3.3. Comparison of Delay Time Based on Preoperative CT Performance

Of the 414 patients, 114 (27.5%) underwent CT first before emergency surgery, while 300 (72.5%) underwent immediate laparotomy. As shown in Table 3 (unweighted), the CT-first group had significantly longer ED-to-OR times (81.0 ± 23.7 vs. 48.4 ± 23.2 min; *p* < 0.001). Patients who underwent CT first also presented with a more favorable baseline status, including a higher median GCS (14 vs. 12, *p* < 0.001), lower 4-h RBC transfusion requirements (8.5 ± 5.0 vs. 13.2 ± 6.9 units, *p* < 0.001), and lower hemorrhagic mortality (6.1% vs. 21.3%, *p* < 0.001). These findings suggest that CT-first was preferentially performed in patients with more stable physiology and less severe hemorrhage.

After IPTW to adjust for confounding factors, most baseline differences between groups were balanced (Table 3, post-weighting). For example, the difference in 4-h RBC transfusion volume was no longer significant (*p* = 0.156). However, the delay associated with preoperative CT remained evident, with mean ED-to-OR times of 80.4 min for the CT-first group and 48.7 min for the immediate laparotomy group (*p* < 0.001). Hemorrhagic mortality was 14.3% in the CT-first group and 10.3% in the immediate laparotomy group, and this difference was attenuated and no longer statistically significant (*p* = 0.542).

Residual imbalances (age, RTS, AIS-head, AIS-thorax, AIS-external, ISS, and transfusion volumes) remained above the 0.10 threshold after IPTW, as illustrated in the Love plot (Supplementary Figure S1). Therefore, we performed a prespecified AIPW analysis to address these residual imbalances and enhance robustness to model misspecification. In this analysis, the estimated risk of hemorrhagic death was 25.2% for the CT-first group and 17.0% for the immediate laparotomy group (risk difference 8.2%; 95% CI, −11.3% to +43.7%), with a risk ratio of 1.48 (95% CI, 0.32–3.60). These findings were consistent with the IPTW results, confirming no statistically significant difference between the two groups.

**Figure 2:**
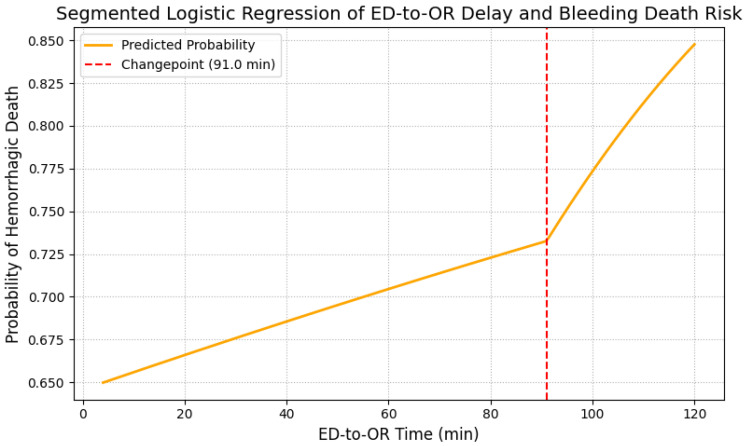
Segmented logistic regression analysis of ED-to-OR delay and hemorrhagic death risk. Segmented logistic regression model of the association between ED-to-OR time and hemorrhagic mortality. The yellow curve represents the estimated probability of hemorrhagic death, and the red dashed line indicates the estimated changepoint (91 min; bootstrap 95% CI, 62.0–97.6 min). ED, emergency department; OR, operating room.

Figure 2 presents the relationship between the ED-to-OR time and hemorrhagic death risk, assessed with a segmented logistic regression model. In this model, a changepoint was observed at approximately 91 min (bootstrap 95% CI 62.0–97.6 min), beyond which the slope of mortality risk increased more steeply. Specifically, when the ED-to-OR interval exceeded approximately 90 min, the probability of hemorrhagic death showed a steeper increase. The yellow curve depicts the estimated hemorrhagic mortality over time, as modeled by segmented logistic regression, while the red dashed line indicates the estimated change point. Individual patient scatter data were omitted to emphasize the risk trends of the model. These results indicate that in this cohort, the estimated risk of hemorrhagic death increased more steeply when ED-to-OR time exceeded approximately 90 min. This observational association underscores the importance of minimizing surgical delays, although the identified changepoint should be interpreted as exploratory and not as a definitive causal threshold.

## 4. Discussion

This multicenter study demonstrated that trauma patients undergoing emergency laparotomy for abdominal injury who died of hemorrhage tended to have longer ED-to-OR times. Segmented logistic regression identified a changepoint at approximately 90 min, beyond which the risk of mortality rose sharply. Although preoperative CT was associated with surgical delay, it was not independently linked to increased hemorrhagic mortality after adjustment for physiologic severity and transfusion requirements.

Patients who died from hemorrhage presented with more severe clinical derangements at ED arrival, including tachycardia, lower GCS scores, and higher transfusion requirements, reflecting profound hemodynamic compromise. These findings are consistent with prior reports and emphasize the prognostic importance of physiologic status on arrival [12,13]. The higher abdominal AIS observed among hemorrhagic deaths further corroborates the role of abdominal injury severity, in line with the findings of Karmy-Jones et al. [14]. Importantly, although crude comparisons showed no significant difference in ED-to-OR time between survivors and non-survivors, multivariable analysis identified surgical delay as an independent predictor of mortality. This discrepancy likely reflects confounding, as more unstable patients underwent expedited surgery, thereby attenuating unadjusted group differences. After controlling for physiologic severity, the association between surgical delay and hemorrhagic death remained significant.

The time-dependent relationship between surgical delay and patient outcomes has been highlighted in numerous studies. Clarke et al. found that survival decreased significantly when laparotomy was delayed beyond 90 min [3], while Barbosa et al. demonstrated a 50% increase in the odds of death for every 10-min delay [6]. Our study further quantified this risk using segmented regression and revealed a turning point at approximately 91 min, beyond which the risk of hemorrhagic death increased disproportionately. This finding supports the hypothesis that a finite window exists during which hemorrhage control can significantly alter the trajectory of an exsanguinating shock [15,16].

However, not all investigations have demonstrated a direct association between shorter admission-to-surgery intervals and improved survival. Grisel et al. (2024) analyzed over 41,000 patients with penetrating abdominal trauma using the ACS TQIP database and paradoxically found that at the patient level, each additional minute of time-to-OR was associated with a 1.5% decrease in the odds of in-hospital mortality (OR: 0.985; 95% CI: 0.981–0.989; *p* < 0.001), although no significant differences were observed across facilities [17]. Similarly, in a 10-year multicenter study from Taiwan, Tsai et al. (2024) reported that the waiting time to emergency surgery was not significantly associated with in-hospital mortality, ICU admission, or prolonged hospitalization, with some subgroups even showing improved survival with longer intervals [18]. Both groups of authors emphasized that these counterintuitive findings are likely attributable to bias—particularly the fact that the most severely injured patients are triaged to the operating room most rapidly, thereby inflating mortality in the “short-interval” cohort—and to the confounding influence of presurgical stabilization measures such as transfusion, hemostatic agents, and ventilatory support. Taken together, these contrasting results suggest that while our analysis identified a clear inflection point around 90 min beyond which hemorrhagic mortality rises steeply, time-to-surgery must be interpreted in the broader context of patient selection, physiologic stabilization, and institutional workflows.

In addition to surgical timing, transfusion volume within the first 4 h was strongly associated with hemorrhagic death, but this should be interpreted as a marker of bleeding severity rather than a causal determinant. This interpretation is consistent with the prior trauma literature, in which early transfusion requirements have been regarded as surrogate markers of hemorrhage severity and associated with worse outcomes [19,20,21]. The protective effect of higher GCS scores observed in our study supports the concept that preserved neurological function reflects better perfusion and lower risk of death from hemorrhagic causes [22].

Our preoperative CT findings require further investigation. Although CT was associated with delays to the OR, it was not independently associated with increased mortality after statistical adjustment. This contrasts with earlier studies which reported poorer outcomes among hypotensive trauma patients undergoing preoperative CT [23]. Neal et al. emphasized that CT in unstable patients may delay definitive hemorrhage control [7], and similar delays have been linked to adverse outcomes in other studies [24]. A likely explanation for the discrepancy is selection bias: in our cohort, CT was almost exclusively performed in patients with more stable physiology, as reflected by higher GCS scores and lower transfusion requirements. Accordingly, while these findings suggest that CT may be permissible in carefully selected stable patients and could aid surgical planning [25,26], they should not be taken as evidence that CT is safe in unstable patients.

Several trauma guidelines discourage the routine use of CT in unstable patients requiring emergent laparotomy [27]. In contrast, the role of CT in hemodynamically stable trauma patients remains an important consideration in clinical decision-making. Poletti et al. CT especially modern multi-slice CT offers unparalleled diagnostic accuracy in detecting visceral organ injuries in hemodynamically stable trauma victims. They emphasized that CT not only surpasses ultrasound in sensitivity and reproducibility but also enables comprehensive assessment of multiple body regions in a single session, thereby informing timely and tailored management strategies [28]. Furthermore, Yamamoto et al. demonstrated that even in severely injured trauma patients, when managed in an all-in-one resuscitation room equipped with streamlined protocols, whole-body CT could be performed within 10 min of arrival and was associated with reduced in-hospital mortality, suggesting that under such conditions the risks of CT are minimized and the benefits can outweigh potential delays [29].

Change-point analysis in our study identified an exploratory quantitative threshold, supporting the concept of the “golden hour” in trauma resuscitation. Earlier studies by Cannon et al. and Duchesne et al. emphasized the importance of initiating hemorrhage control within 60–90 min of arrival [30,31]. The steep rise in mortality beyond 90 min in our cohort suggests a potential inflection point at which the physiologic reserve may be critically compromised. However, given the observational design of this study, the apparent changepoint may partly reflect system-level workflow characteristics (e.g., ED triage, CT decision-making and transport logistics, operating-room availability and team mobilization and preoperative stabilization practices) and patient-selection patterns (e.g., relatively stable patients preferentially receiving CT or additional resuscitation while the most unstable proceed directly to laparotomy). Accordingly, the 91-min estimate should be regarded as a data-driven, exploratory association rather than a strict physiologic threshold or proof of causality.

Institutional variability in ED-to-OR time continues to pose a major challenge in trauma systems. Prior studies have highlighted substantial inter-institutional disparities in trauma system efficiency and underscored the need for time-sensitive protocols [32,33]. Studies have linked improved outcomes to rapid operating-room access, parallel resuscitation and surgical preparation, and coordinated trauma team activation in both military and civilian settings [34,35]. Our findings support the implementation of targeted quality improvement strategies to minimize surgical delays, particularly for high-risk patients with physiologic instability or severe ongoing hemorrhage.

This study has some limitations. First, as a retrospective analysis, this study is inherently prone to selection bias and residual confounding. Because patient inclusion and data collection were not prospectively randomized or standardized, important prognostic factors may have been unevenly distributed or not captured, limiting the strength of causal inference. Although retrospective designs leverage real-world clinical data, they rely on records not originally intended for research, which introduces interpretative challenges that must be acknowledged when drawing conclusions [36]. In particular, early transfusion reflects active bleeding and hemorrhage severity; thus, its association with mortality should be interpreted only as a marker of hemorrhage severity rather than as a causal determinant in this observational study.

Second, the classification of hemorrhagic death relied on a multidisciplinary panel review rather than autopsy, which may have introduced subjective bias and misclassification. Although structured clinical judgment provides some consistency, prior studies have demonstrated substantial discrepancies between clinically determined and autopsy-confirmed causes of trauma-related death. In trauma patients with multiple injuries and physiologic derangements, clinical assessments alone may be insufficiently accurate to establish the primary cause of death, potentially affecting quality improvement efforts [37].

Third, although IPTW was used to balance baseline covariates between the CT and immediate surgery groups, treatment allocation was inherently driven by prognosis-related factors such as hemodynamic stability and transfusion requirements. Thus, unmeasured confounding related to prognosis likely remains even after weighting, thereby limiting the causal interpretation of our findings. Importantly, no statistical method can completely eliminate systematic treatment allocation bias in observational studies, and our results should therefore be interpreted as associations rather than causal effects.

Fourth, the comparison between CT-first and immediate laparotomy is subject to immortal time bias, as patients must survive and remain sufficiently stable to undergo CT. In our analysis, CT was not modeled as a time-dependent exposure, and some baseline covariates may have been influenced by events occurring after the imaging decision. Consequently, the observed association between CT use and mortality should be interpreted with caution, given the potential for bias. To more accurately infer causality, future studies would require a time-dependent Cox model or a target-trial emulation that restricts analyses to covariates measured before treatment assignment.

Fifth, the generalizability of our findings is limited by the study setting, which involved only two trauma centers within a single national healthcare system. These centers may have greater resources and faster operative capabilities than smaller or decentralized institutions, where outcomes could differ. Therefore, broader validation through multicenter or international studies is required to support wider application of these findings

In summary, this multicenter retrospective analysis found that longer ED-to-OR intervals were associated with higher hemorrhagic mortality, with segmented regression identifying a steeper rise in risk beyond approximately 90 min. These findings are consistent with the principle that ‘time is blood’ in exsanguinating trauma. Although preoperative CT was associated with procedural delays, it was not independently associated with hemorrhagic death after adjustment, reflecting its selective use in patients with greater physiologic stability. Together, these results describe observational associations in current practice, rather than prescriptive recommendations, regarding the timing of surgery and the use of preoperative CT.

Randomized controlled trials would represent the methodological gold standard to establish causality, but such studies are ethically and practically infeasible in critically bleeding trauma patients. Therefore, prospective multicenter registries and advanced time-dependent analyses represent feasible approaches to further validate these associations and inform future clinical decision-making.

## Figures and Tables

**Table 1:** Comparison of Demographics and Outcomes Between Survivors and Hemorrhagic Death.

	Overall Group (*n* = 442)	Survivors (*n* = 343)	All-Cause Death (*n* = 99)	*p*	Hemorrhagic Death (*n* = 71)	*p*
Male, *n* (%)	323 (73.1%)	245 (71.4%)	78 (78.8%)	0.942	56 (78.9%)	0.966
Age	48.9 ± 17.2	48.5 ± 16.8	50.1 ± 18.4	0.424	49.0 ± 17.9	0.813
Blunt	339 (76.7%)	248 (72.3%)	91 (91.9%)	0.005	64 (90.1%)	0.041
Systolic Blood Pressure	109.5 ± 28.4	110.3 ± 27.8	107.3 ± 30.2	0.360	108.0 ± 30.1	0.541
Pulse Rate	83.5 ± 27.8	82.1 ± 27.4	87.6 ± 28.9	0.088	90.4 ± 26.9	0.022
Glasgow Coma Scale	13 (8, 15)	14 (10, 15)	7 (3, 12)	<0.001	7 (3, 11)	<0.001
Revised Trauma Score	10.5 ± 1.5	10.8 ± 1.3	9.1 ± 1.8	<0.001	9.3 ± 1.8	<0.001
Abbreviated Injury Score						
Head	1.0 ± 1.5	1.0 ± 1.5	0.9 ± 1.8	0.581	0.3 ± 1.0	<0.001
Face	0.4 ± 0.7	0.4 ± 0.8	0.2 ± 0.6	0.022	0.1 ± 0.5	0.005
Thorax	2.3 ± 1.6	2.4 ± 1.5	2.0 ± 1.8	0.023	1.9 ± 1.9	0.006
Abdomen	3.6 ± 1.0	3.5 ± 0.9	3.9 ± 1.2	0.003	4.1 ± 1.1	<0.001
External	0.8 ± 0.6	0.8 ± 0.6	0.6 ± 0.5	0.012	0.6 ± 0.6	0.001
Injury Severity Score	30.9 ± 13.7	30.2 ± 13.4	33.3 ± 14.4	0.052	32.6 ± 14.4	0.170
First 4-h RBC transfusion (units)	11.9 ± 6.7	10.3 ± 5.8	16.8 ± 7.1	<0.001	18.0 ± 7.0	<0.001
First 24-h RBC transfusion (units)	14.7 ± 11.4	12.4 ± 8.8	22.0 ± 15.0	<0.001	23.1 ± 12.9	<0.001
ED-to-OR time (min)	57.4 ± 27.5	56.5 ± 26.2	60.1 ± 31.2	0.252	62.3 ± 31.9	0.107

This study is observational; therefore, baseline imbalances reflect non-randomized group allocation. Values are presented as mean ± SD or median (IQR) as appropriate. ED, emergency department; OR, operating room; RBC, red blood cell; h, hour.

**Table 2:** Multivariable Logistic Regression Analysis for Prediction of Hemorrhagic Death.

	Odds Ratio	95% CI	*p*
ED-to-OR time	1.018	1.007–1.030	0.002
Glasgow Coma Scale	0.799	0.739–0.864	<0.001
RBC transfusion first 4 h	1.176	1.118–1.237	<0.001
Injury Severity Score	0.982	0.959–1.006	0.143
Age	1.014	0.995–1.034	0.159
Systolic Blood Pressure	1.001	0.990–1.013	0.813

ED, emergency department; OR, operating room; CI, confidence interval.

**Table 3:** Clinical Comparison of Immediate Surgery and CT-First Groups With and Without IPTW Adjustment.

	Overall (*n* = 414)	Unweighted				Weighted			
		Immediate Laparotomy (*n* = 300)	CT-First (*n* = 114)	*p*	SMD	Immediate Laparotomy (*n* = 199)	CT-First (*n* = 121.8)	*p*	SMD
ED-to-OR time (min)	57.4 ± 27.5	48.4 ± 23.2	81.0 ± 23.7	<0.001		48.7 ± 22.6	80.4 ± 25.3	<0.001	
Age	48.9 ± 17.2	48.0 ± 17.7	51.1 ± 15.8	0.085	0.20	49.9 ± 17.9	51.9 ± 13.3	0.242	0.30
Systolic Blood Pressure	109.5 ± 28.4	109.2 ± 28.7	110.3 ± 27.7	0.733	0.03	107.7 ± 31.1	102.6 ± 30.5	0.146	0.04
Pulse Rate	83.5 ± 27.8	83.8 ± 28.7	82.5 ± 25.5	0.649	0.06	81.0 ± 31.5	79.4 ± 28.6	0.630	0.02
Glasgow Coma Scale	13.0 (8.0, 15.0)	12.0 (7.0, 14.0)	14.0 (12.0, 15.0)	<0.001	0.49	11.7 ± 3.7	11.3 ± 4.4	<0.001	0.05
Revised Trauma Score	10.5 ± 1.5	10.4 ± 1.5	10.8 ± 1.5	0.039	0.38	7.7 ± 2.5	7.3 ± 2.7	0.102	0.10
Abbreviated Injury Score									
Head	1.0 ± 1.5	0.9 ± 1.5	1.1 ± 1.6	0.216	0.12	1.5 ± 1.8	1.3 ± 1.4	0.221	0.13
Face	0.4 ± 0.7	0.3 ± 0.7	0.4 ± 0.8	0.244	0.14	0.3 ± 0.6	0.3 ± 0.5	0.754	0.07
Thorax	2.3 ± 1.6	2.3 ± 1.6	2.4 ± 1.4	0.947	0.02	2.1 ± 1.9	1.9 ± 1.4	0.321	0.16
Abdomen	3.6 ± 1.0	3.6 ± 1.1	3.6 ± 0.8	0.811	0.02	3.8 ± 1.1	3.6 ± 0.9	0.045	0.09
External	0.8 ± 0.6	0.7 ± 0.5	0.8 ± 0.6	0.084	0.11	0.8 ± 0.7	0.7 ± 0.5	0.310	0.30
Injury Severity Score	30.9 ± 13.7	31.2 ± 14.1	30.2 ± 12.5	0.485	0.10	33.1 ± 15.0	32.5 ± 13.0	0.712	0.12
First 4-h RBC transfusion (units)	11.9 ± 6.7	13.2 ± 6.9	8.5 ± 5.0	<0.001	0.79	14.0 ± 7.6	12.4 ± 6.8	0.156	0.33
First 24-h RBC transfusion (units)	14.7 ± 11.4	16.2 ± 12.1	10.8 ± 8.2	<0.001	0.53	16.8 ± 12.2	14.1 ± 9.8	0.083	0.40
Hemorrhagic Death	71 (17.2%)	64 (21.3%)	7 (6.1%)	<0.001		20.5 (10.3%)	17.4 (14.3%)	0.542	

Values are presented as mean ± SD or median (IQR) as appropriate. Bleeding by Death proportion was compared using the z-test for proportions. SMD, standardized mean difference; values >0.10 indicate residual imbalance after weighting. IPTW, inverse probability of treatment weighting; ED, emergency department; OR, operating room; RBC, red blood cell.

## Data Availability

The data presented in this study are available on request from the corresponding author. The data are not publicly available due to privacy and ethical restrictions.

## Supplementary Materials

The following supporting information can be downloaded at https://www.mdpi.com/article/10.3390/jcm14197020/s1. File S1: STROBE Statement—Checklist of items that should be included in reports of cohort studies; Figure S1: Love plot demonstrating covariate balance before and after weighting.

## Abbreviations

The following abbreviations are used in this manuscript:

ED	Emergency Department
OR	Operating Room
CT	Computed Tomography
GCS	Glasgow Coma Scale
RTS	Revised Trauma Score
AIS	Abbreviated Injury Scale
ISS	Injury Severity Score
PRBCs	Packed Red Blood Cells
KTDB	Korea Trauma Data Bank
IPTW	Inverse Probability of Treatment Weighting
STROBE	Strengthening the Reporting of Observational Studies in Epidemiology
OR	Odds Ratio
CI	Confidence Interval

## References

[1] Lozano R., Naghavi M., Foreman K., Lim S., Shibuya K., Aboyans V., Abraham J., Adair T., Aggarwal R., Ahn S.Y. (2012). Global and regional mortality from 235 causes of death for 20 age groups in 1990 and 2010: A systematic analysis for the Global Burden of Disease Study 2010. Lancet.

[2] Spahn D.R., Cerny V., Coats T.J., Duranteau J., Fernández-Mondéjar E., Gordini G., Stahel P.F., Hunt B.J., Komadina R., Neugebauer E. (2007). Management of bleeding following major trauma: A European guideline. Crit. Care.

[3] Clarke J.R., Trooskin S.Z., Doshi P.J., Greenwald L., Mode C.J. (2002). Time to laparotomy for intra-abdominal bleeding from trauma does affect survival for delays up to 90 minutes. J. Trauma.

[4] Malinoski D.J., Patel M.S., Yakar D.O., Green D., Qureshi F., Inaba K., Brown C.V.R.M., Salim A. (2010). A diagnostic delay of 5 hours increases the risk of death after blunt hollow viscus injury. J. Trauma.

[5] Fakhry S.M., Brownstein M., Watts D.D., Baker C.C., Oller D. (2000). Relatively short diagnostic delays (<8 hours) produce morbidity and mortality in blunt small bowel injury: An analysis of time to operative intervention in 198 patients from a multicenter experience. J. Trauma.

[6] Barbosa R.R., Rowell S.E., Fox E.E., Holcomb J.B., Bulger E.M., Phelan H.A., Alarcon L.H., Myers J.G., Brasel K.J., Muskat P. (2013). Increasing time to operation is associated with decreased survival in patients with a positive FAST examination requiring emergent laparotomy. J. Trauma Acute Care Surg..

[7] Neal M.D., Peitzman A.B., Forsythe R.M., Marshall G.T., Rosengart M.R., Alarcon L.H., Billiar T.R., Sperry J.L. (2011). Over reliance on computed tomography imaging in patients with severe abdominal injury: Is the delay worth the risk?. J. Trauma.

[8] Mitra B., Mori A., Cameron P.A., Fitzgerald M., Paul E., Street A. (2010). Fresh frozen plasma use during massive blood transfusion in trauma resuscitation. Injury.

[9] Kashuk J.L., Moore E.E., Johnson J.L., Haenel J.R., Wilson M., Moore J.B., Cothren C.C., Biffl W.L., Banerjee A., Sauaia A. (2008). Postinjury life-threatening coagulopathy: Is 1:1 fresh frozen plasma to packed red blood cells the answer?. J. Trauma.

[10] Meyer D.E., Cotton B.A., Fox E.E., Stein D., Holcomb J.B., Cohen M., Inaba K., Rahbar E. (2018). A comparison of resuscitation intensity and critical administration threshold in predicting early mortality among bleeding patients: A multicenter validation in 680 major transfusion patients. J. Trauma Acute Care Surg..

[11] Chiara O., Cimbanassi S., Pitidis A., Vesconi S. (2006). Preventable trauma deaths: From panel review to population-based studies. World J. Emerg. Surg..

[12] Demetriades D., Kuncir E.J., Velmahos G.C. (2004). Outcomes and prognostic factors in head injuries with an admission Glasgow Coma Scale score of 3. Arch. Surg..

[13] Shapiro M.B., Jenkins D.H., Schwab C.W., Rotondo M.F. (2000). Damage control: Collective review. J. Trauma.

[14] Karmy-Jones R., Jurkovich G.J., Nathens A.B., Shatz D.V., Brundage S., Wall M.J., Engelhardt S., Hoyt D.B., Holcroft J., Knudson M.M. (2001). Timing of urgent thoracotomy for hemorrhage after trauma: A multicenter study. Arch. Surg..

[15] Chang R., Fox E.E., Greene T.J., Hinckley W.R., Holcomb J.B., Scalea T.M., Stein D.M., Bulger E.M., Schreiber M.A., Alarcon L.H. (2019). Earlier time to hemostasis is associated with decreased mortality and rate of complications: Results from the PROMMTT study. J. Trauma Acute Care Surg..

[16] Morrison J.J., Galgon R.E., Jansen J.O., Cannon J.W., Rasmussen T.E., Eliason J.L. (2016). A clinical series of resuscitative endovascular balloon occlusion of the aorta for hemorrhage control and resuscitation. Lancet.

[17] Grisel B., Gordee A., Kuchibhatla M., Ginsberg Z., Agarwal S., Haines K. (2024). Outcomes by time-to-OR for penetrating abdominal trauma patients. Am. J. Emerg. Med..

[18] Tsai C.H., Wu M.Y., Chien D.S., Lin P.C., Chung J.Y., Liu C.Y., Tzeng I.S., Hou Y.T., Chen Y.L., Yiang G.T. (2024). Association between time to emergent surgery and outcomes in trauma patients: A 10-year multicenter study. Medicina.

[19] Holcomb J.B., Wade C.E., Michalek J.E., Chisholm G.B., Zarzabal L.A., Schreiber M.A., Gonzalez E.A., Pomper G.J., Perkins J.G., Spinella P.C. (2005). Increased plasma and platelet to red blood cell ratios improves outcome in 466 massively transfused civilian trauma patients. J. Trauma.

[20] Sperry J.L., Ochoa J.B., Gunn S.R., Alarcon L.H., Minei J.P., Cuschieri J., Rosengart M.R., Maier R.V., Billiar T.R., Peitzman A.B. (2008). An FFP:PRBC transfusion ratio ≥1:1.5 is associated with a lower risk of mortality after massive transfusion. J. Trauma.

[21] Rahbar E., Cardenas J.C., Matijevic N., Cotton B.A., Holcomb J.B. (2015). Early platelet dysfunction: An unrecognized role in the acute coagulopathy of trauma. J. Trauma Acute Care Surg..

[22] Iyanna N., Vavilala M.S., Bourdette D., Dagal A., Idris A., Christie S.A., Bulger E.M., Cohen D.J., He H., Yealy D.M. (2024). Early Glasgow Coma Scale Score and Prediction of Traumatic Brain Injury: A Secondary Analysis of Three Harmonized Prehospital Randomized Clinical Trials. Prehosp. Emerg. Care.

[23] Bilello J.F., Davis J.W., Lemaster D., Townsend R.N., Parks S.N., Sue L.P., Kaups K.L., Groom T., Egbalieh B. (2011). Prehospital hypotension in blunt trauma: Identifying the “crump factor”. J. Trauma.

[24] Tinkoff G., Esposito T.J., Reed J., Kilgo P., Fildes J., Pasquale M., Meredith J.W. (2008). American Association for the Surgery of Trauma Organ Injury Scale I: Spleen, liver, and kidney, validation based on the National Trauma Data Bank. J. Am. Coll. Surg..

[25] Raza M., Abbas Y., Devi V., Prasad K.V.S., Rizk K.N., Nair P.P. (2013). Non operative management of abdominal trauma—A 10 years review. World J. Emerg. Surg..

[26] Poletti P.A., Mirvis S.E., Shanmuganathan K., Killeen K.L., Coldwell D.M. (2002). CT criteria for management of blunt liver trauma: Correlation with angiographic and surgical findings. Eur. Radiol..

[27] Hoff W.S., Holevar M., Nagy K.K., Patterson L., Young J.S., Arrillaga A., Najarian M.P., Valenziano C.P. (2002). Practice Management Guidelines for the Evaluation of Blunt Abdominal Trauma: The EAST Practice Management Guidelines Work Group. J. Trauma.

[28] Poletti P.-A., Wintermark M., Schnyder P., Becker C.D. (2002). Traumatic injuries: Role of imaging in the management of the polytrauma victim (conservative expectation). Eur. Radiol..

[29] Yamamoto R., Suzuki M., Funabiki T., Sasaki J. (2023). Immediate CT after hospital arrival and decreased in-hospital mortality in severely injured trauma patients. BJS Open.

[30] Cannon J.W., Khan M.A., Raja A.S., Cohen M.J., Como J.J., Cotton B.A., Dubose J.J., Fox E.E., Inaba K., Rodriguez C.J. (2017). Damage control resuscitation in patients with severe traumatic hemorrhage: A practice management guideline from the Eastern Association for the Surgery of Trauma. J. Trauma Acute Care Surg..

[31] Duchesne J.C., McSwain N.E., Cotton B.A., Hunt J.P., Dellavolpe J., Lafaro K., Marr A.B., Gonzalez E.A., Phelan H.A., Bilski T. (2010). Damage control resuscitation: The new face of damage control. J. Trauma.

[32] Callcut R.A., Kornblith L.Z., Conroy A.S., Robles A.J., Meizoso J.P., Namias N., Meyer D.E., Haymaker A., Truitt M.S., Agrawal V. (2019). The why and how our trauma patients die: A prospective Multicenter Western Trauma Association study. J. Trauma Acute Care Surg..

[33] Lane S., Nahmias J., Lekawa M., Fox J.C., Chandwani C., Lotfipour S., Grigorian A. (2024). Comparison of Emergency Department Disposition Times in Adult Level I and Level II Trauma Centers. West. J. Emerg. Med..

[34] Blackbourne L.H., Baer D.G., Eastridge B.J., Kheirabadi B.S., Kragh J.F., Cap A.P., Dubick M.A., Morrison J.J., Midwinter M.J., Butler F.K. (2012). Military medical revolution: Prehospital combat casualty care. J. Trauma Acute Care Surg..

[35] Apodaca A., Olson C.M., Bailey J., Butler F., Eastridge B.J., Kuncir E. (2013). Performance Improvement Evaluation of Forward Aeromedical Evacuation Platforms in Operation Enduring Freedom. J. Trauma Acute Care Surg..

[36] Talari K., Goyal M. (2020). Retrospective studies—Utility and caveats. J. R. Coll. Physicians Edinb..

[37] Esposito T.J., Sanddal T., Sanddal N., Whitney J. (2012). Dead men tell no tales: Analysis of the use of autopsy reports in trauma system performance improvement activities. J. Trauma Acute Care Surg..

