# Supplementary figures and images for: Association of Preoperative Imaging and Surgical Delay with Hemorrhagic Mortality in Abdominal Trauma: A Retrospective Multicenter Study
Source: J Clin Med. 2025 Oct 3;14(19):7020. doi: 10.3390/jcm14197020 (PMC12524961; doi:10.3390/jcm14197020)

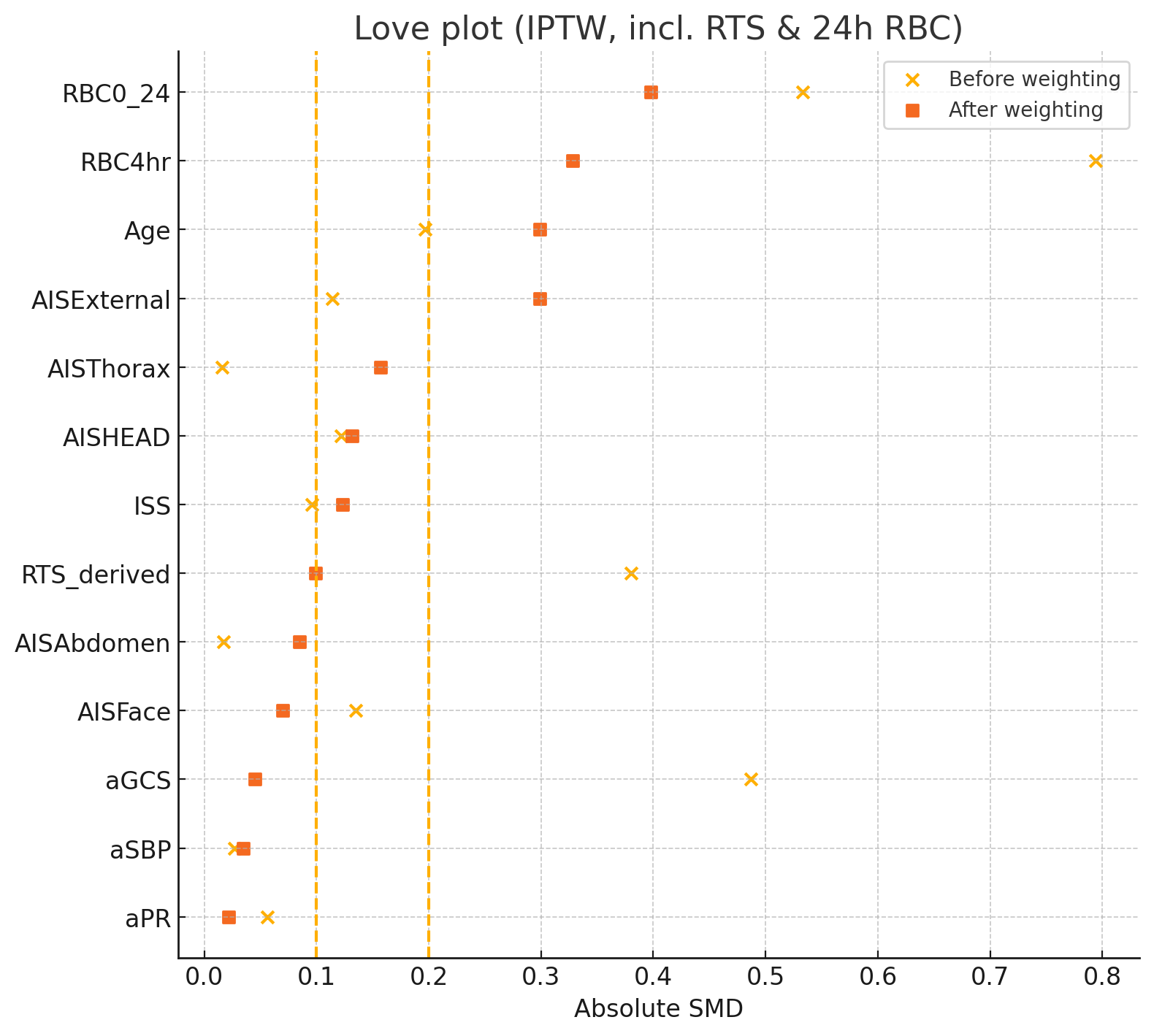

Supplement: Supplementary file 1 [file jcm-14-07020-s001.zip › Figure S1.png]
